# Supplementary material for: A link between smoking behaviors and the risk of hidradenitis suppurativa in diabetic patients
Source: PLoS One. 2025 Jun 5;20(6):e0325357. doi: 10.1371/journal.pone.0325357 (PMC12140424; doi:10.1371/journal.pone.0325357)
Supplement: Attachment — Submitted filename: Response to Reviewers.docx [file pone.0325357.s002.docx]

To

Editor of PLOS ONE

Submission ID d914ed04d190a2b4

"Hidradenitis suppurativa (HS) in diabetes mellitus patients: A link between alterations in smoking intensity and the risk of developing HS"

Thank you very much for your kind editorial letter.

We have attempted to carefully and thoroughly address all concerns raised by the editors and reviewers. With the help of your suggestions, we believe our manuscript has significantly improved.

We trust that we therewith have fulfilled all the Editor’s and Reviewer’s requests.

Thank you very much for your consideration.

Sincerely,

Ji Hyun Lee, MD. PhD

Department of Dermatology, Seoul St. Mary`s Hospital, College of Medicine, The Catholic University of Korea, Seoul, 06591, Korea.

Tel: +82-2-2258-1398

Fax: +82-70-7382-1380

E-mail: [yiji1@hanmail.net](mailto:yiji1@hanmail.net)

**Responses to Editor**

Comments**(C)** from the Editor:

**Journal Requirements:**

**C 1.** When submitting your revision, we need you to address these additional requirements.

Please ensure that your manuscript meets PLOS ONE's style requirements, including those for file naming. The PLOS ONE style templates can be found at <https://journals.plos.org/plosone/s/file?id=wjVg/PLOSOne_formatting_sample_main_body.pdf> and <https://journals.plos.org/plosone/s/file?id=ba62/PLOSOne_formatting_sample_title_authors_affiliations.pdf>

**C 2.** In the online submission form, you indicated that the data in this study was obtained from KNHIS where license may apply. Further enquiries can be directed to the corresponding author.

All PLOS journals now require all data underlying the findings described in their manuscript to be freely available to other researchers, either a. In a public repository, b. Within the manuscript itself, or c. Uploaded as supplementary information.
This policy applies to all data except where public deposition would breach compliance with the protocol approved by your research ethics board. If your data cannot be made publicly available for ethical or legal reasons (e.g., public availability would compromise patient privacy), please explain your reasons on resubmission and your exemption request will be escalated for approval.

**Response:** The data used in this study were obtained from a national database **with restricted access for a specific period**, making it difficult to publicly share all datasets. However, if additional information is needed, it can be requested and verified through the corresponding author.

Thank you for your feedback and for outlining the requirements regarding data accessibility for our manuscript.

In compliance with PLOS’ Data Policy, we have identified and included a **non-author institutional point of contact** to ensure long-term data availability. The details are as follows:

- **Institutional Point of Contact:** Research Office
- **Contact Information:** dermaseoul@gmail.com

We hope that this satisfies your requirements. Should you require any additional information, please do not hesitate to contact us. Thank you for your guidance.

**Responses to Reviewer**

Reviewer(s)' Comments to the Author:
**Reviewer: 1**

Comments to the Author
Thank you for inviting me to review this paper. The authors have investigated the association of alterations in smoking intensity and the risk of developing HS in patients with DM. Overall, the association of smoking and HS has been previously studied. Thus, the topic is not a novel one. However, the finding regarding BMI is interesting. The method section needs to be improved as it requires more detail.

My comments are the followings:

**C 1.** -line 39: compared to which population?

**Response:** It was compared to nonsmokers. We added this in **P 2 L 42.** The revised text is highlighted in red.

**C 2.** -line 40: please clarify that you are discussing smoking.

**Response:** We added it in **P 2 L 43-44.**

**C 3.** -line 47: which year?

**Response:** Between 1968 and 2008, 268 HS cases were identiﬁed leading to a mean incidence of 6.0 per 100 000 person-years with a twofold incidence between the extremities of the period (4 to 10 per 100 000 person-years) (**Ref. #3. Vazquez BG, et al. J Invest Dermatol 2013; 133:97–103.; Ref. #1. Zouboulis CC, et al. J Eur Acad Dermatol Venereol. 2015;29(4):619-44.)**. By postulating a duration of active HS being a maximum of about 20 years for a given subject, the incidence can be extrapolated to prevalence = incidence * duration of the disease. Accordingly, the prevalence would be:

• Max: 10/100 000 years * 20 years = 200/100 000 = 0.20%.

• Min: 4/100 000 years * 20 years = 80/100 000 = 0.08%.

This conclusion is supported by expert opinion. (**Ref. #1. Zouboulis CC, et al. J Eur Acad Dermatol Venereol. 2015;29(4):619-44.)**

A recent American prevalence study based on a health claims database estimated HS prevalence to be 0.05% **(Cosmatos I, Matcho A, Weinstein R et al. Analysis of patient claims data to determine the prevalence of hidradenitis suppurativa in the United States. J Am Acad Dermatol 2013; 68: 412–419.)**. Two studies from Europe reported a prevalence of 1% **(Ref. #27. Revuz JE, et al. J Am Acad Dermatol 2008; 59: 596–601.; Ref. #4. Jemec GB, et al. J Am Acad Dermatol 1996; 35: 191–194.),** and according to Jemec GB, et al., the prevalence rates have ranged from 0.00033 to **4%.**

- We added these contents and references in **P4 L54.**

**C 4.** -did the authors exclude patients using anti-diabetic medications for reasons other than DM?

**Response:** Yes, we excluded patients using anti-diabetic drugs for indications other than diabetes, as we selected patients meeting the following criteria: (1) who had been diagnosed with DM under ICD-10 codes “E11–14” **and** used anti-diabetic drugs, or (2) who met the DM diagnostic criterion of a fasting plasma glucose level equal to or greater than 126 mg/dL during a health examination (in **P6 L107-110**, “**Definition of disease**”).

**C 5.** -add more details on blood sample collection and measurement methods.

**Response:** We added these contents and references in **P7 L137-139.**

**C 6.** -add more details on height and weight measurement for BMI calculation.

**Response:** We added these contents and references in **P7 L139-141.**

**C 7.** -cite the reference associated with physical activity categories.

**Response:** We added references in **P7 L144-146.**

**C 8.** - line 165: which figure? also add numerical values.

**Response:** Since new figures have been added, and the previous “Figure” has been renamed as “**Figure 3**”.

**C 9.** - mention the observational nature of the study as limitation for finding causation.

**Response:** We added references in **P12 L265-266.**

**C 10.** -define abbreviations below the tables.

**Response:** We have added definitions for the abbreviations below the tables.

**C 11.** -what is the possible explanation for finding no association after BMI stratification?

**Response:** The lack of association between changes in smoking intensity and the risk of HS after stratifying by BMI could be attributed to several factors. The limited number of HS events in the study may have reduced the statistical power to detect differences across BMI categories, increasing the likelihood of a Type II error.

Also, if the BMI distribution in the study was relatively homogenous or skewed toward specific categories, it might have limited the ability to observe differences between groups. And the interplay between smoking, BMI, and HS risk is complex and likely involves biological pathways that are not fully elucidated. Factors such as inflammation, hormonal changes, or metabolic syndromes linked to both smoking and BMI might obscure direct associations. Further research with larger and more diverse cohorts is necessary to clarify these findings and explore the interaction between smoking, BMI, and HS risk.

**Reviewer: 2**

Comments to the Author

**C 1.** It is suggested that the manuscript should replace instances of "we" or "our" with phrases such as "current study," "this study," or "present study" to minimize repetition.

**Response:** Thank you for the suggestion. We have made the revisions as recommended and replaced instances of 'we' or 'our' with phrases such as 'current study,' 'this study,' or 'present study' to minimize repetition.

**C 2.** The rationale of the study should be clearly stated at the end of the introduction.
Extensive proofreading and rephrasing are essential.

**Response:** Thank you for your insightful comment. While both diabetes mellitus (DM) and smoking are established risk factors for HS, their combined impact on HS development has not been extensively studied. Smoking, a modifiable behavior, may exacerbate systemic inflammation and metabolic disruptions in DM, potentially heightening the risk of HS. Despite this plausible link, research explicitly exploring this interaction is limited. Therefore, our study aims to investigate the effect of smoking behavior changes on HS risk in DM patients, using a large, nationally representative claims database from Korea. By addressing this gap, we hope to shed light on the role of smoking intensity in HS development and identify modifiable risk factors, particularly in individuals with comorbid DM. Ultimately, we aim to inform strategies for reducing HS risk in this high-risk group. These points have been added to the manuscript on **P4 L 79-82**. Additionally, careful proofreading and rephrasing were done to improve its clarity and quality.

**C 3.** The discussion should be more concise and include more references for better comparison with previous research.
Substantial paraphrasing and thorough proofreading are necessary.

**Response:** Thank you for your valuable comment. We have refined the manuscript through multiple rounds of proofreading and added references, enhancing the discussion content. We hope this will help clarify the points the authors intended to convey in this study.

**C 4.** The manuscript has low quality. It did not address any specific gap they intended to fill. Furthermore, it does not add anything to the subject area compared with other published Material. Therefore, any comments regarding the methodology are equivocal. This manuscript does not fulfill the standards established for the journal to be considered for publication.

**Response:** While the relationship between smoking and HS has been explored in prior studies, our research is distinct in several key aspects: **First**, previous studies have primarily examined smoking status and its association with HS. Our study fills a critical gap by investigating the impact of changes in smoking intensity (e.g., increasing, reducing, or quitting) on the risk of HS, which to our knowledge has not been systematically explored before. This dynamic approach offers new insights into the temporal relationship between smoking behaviors and HS development. **Second,** we leveraged a robust cohort of over 1.7 million participants, with detailed stratification of smoking intensity changes over two time points. The inclusion of a diverse range of smoking behaviors (e.g., sustainers, reducers, increasers, quitters, and starters) allows for a nuanced analysis not previously addressed in smaller, single-center studies. **Third,** this study is one of the few to examine the relationship between smoking and HS in an East Asian population, contributing valuable epidemiological data to a field dominated by Western studies. The findings emphasize the importance of considering ethnic and cultural differences in HS risk factors, offering a basis for future comparative studies. **Lastly,** by demonstrating that smoking continuation and increases in smoking intensity significantly elevate the risk of HS, our findings reinforce the importance of smoking cessation as a modifiable risk factor.

We acknowledge the limitations of our study, such as reliance on self-reported smoking data and the observational design. However, these do not diminish the unique contributions of our research to understanding the modifiable risk factors for HS. We hope this clarification underscores the significance and novelty of our work within the subject area and its potential to inform future research and clinical strategies.

**Reviewer: 3**

Comments to the Author

**C 1**. Conclusion: "This study found an association between smoking and the risk of HS, with implications for targeted interventions and smoking cessation strategies in at-risk populations". The conclusion should be less related to the target group of the study (diabetics).

**Response:** Thank you for your valuable comment. We have revised the conclusion section of the abstract and explicitly specified it for the diabetic group.

**C 2**. Results:
Please provide a flow diagram and report numbers of individuals at each stage of study—eg numbers potentially eligible and give reasons for non-participation at each stage.

**Response:** Thank you for your suggestion. We have created a flow diagram and included it as **Figure 1**, detailing the number of individuals at each stage of the study, including those potentially eligible and the reasons for non-participation at each stage.

**C 3**. Results:
Can medication for diabetes affect the association of Smoking on HS? Is this not examined in this study?

**Response:** Diabetic medications, such as metformin or GLP-1 receptor agonists, have anti-inflammatory properties and they improve glucose metabolism and reduce insulin resistance, which may mitigate systemic metabolic dysregulations linked to HS (**Hambly, R.; Kearney, N.; Hughes, R.; Fletcher, J.M.; Kirby, B. Metformin Treatment of Hidradenitis Suppurativa: Effect on Metabolic Parameters, Inflammation, Cardiovascular Risk Biomarkers, and Immune Mediators. *Int. J. Mol. Sci.* 2023, *24*, 6969.; Krajewski, P.K.; Złotowska, A.; Szepietowski, J.C. The Therapeutic Potential of GLP-1 Receptor Agonists in the Management of Hidradenitis Suppurativa: A Systematic Review of Anti-Inflammatory and Metabolic Effects. *J. Clin. Med.* 2024, *13*, 6292.**). Since HS is a chronic inflammatory condition, these medications could modulate the inflammatory pathways exacerbated by smoking, potentially altering the severity or risk of HS. If diabetic medications are not uniformly distributed among study participants, this variability could confound the observed association between smoking and HS. **So, we adjusted diabetic medication use in analyses** to ensure accurate interpretations.

**C 4**. Results:
The median/mean follow-up time should be mentioned

**Response:** The median follow-up time was 5.2 years, with an IQR of 4.1 to 6.1 years (**P8 L176**).

**C 5**. Results:
In the title of the first table, you must also specify the smoking status as in the second table.

**Response:** Thank you for your comment. We have revised the title of the first table to specify the smoking status, aligning it with the format of the second table.

**C 6**. Results:
In Table 3-4, the p value should be reported according to the number of risk ratio estimates (n=6 estimations) in last column.

**Response:** Thank you for your comment. In Tables 3 and 4, the analysis was conducted using multivariable Cox proportional-hazards models, where a single p-value is reported for the overall effect. This p-value represents the statistical significance of the model's estimates rather than separate significance tests for each risk ratio. Since all risk ratio estimates are derived from the same multivariable model, additional p-values for each estimate were not calculated. To address this, we have added **'not applicable (NA)**' under the p-value column for each estimate to clarify this in the tables. We believe this approach appropriately reflects the results of the analysis and ensures clarity in reporting.

**C 7**. Results:
In table 4, you can also do the trend test

**Response:** Thank you for your suggestion. However, the results were not statistically significant. This lack of significance is likely due to the limited sample size, which may have reduced the power to detect a trend.

**Reviewer: 4**

Comments to the Author

**C 1**. **Suggested title**- A link between smoking behaviours and the risk of Hidradenitis Suppurative in diabetic patients

**Response:** Thank you for your valuable comment. We have revised the title, following your advice.

**C 2**. **Abstract**-is too simplified and does not explore why the team wanted to study HS in DM and link with smoking.

**Response:** We have elaborated on the research objectives and revised the abstract.

**C 3**. **Intro**-intro talks more about HS-DM cause-effect relationship and touched only abit about smoking & HS

**Response:** We have rearranged the paragraphs and added new sentences in **P4 L74-76**. We sincerely appreciate your insightful advice.

**C 4**. Intro
- line 63: Two meta-analyses found a 1.69–3.00 fold increase in the odds of DM in HS populations [10, 23, 24]. (added fold)

**Response:** Thank you for your comment. We have added it.

**C 5**. Intro- line 68: Therefore, we aimed to investigate the effects of changes in smoking on the risk of HS in patients with DM using a large-scale, nationally-representative claims database in Korea. (fragmented sentence, suggest revise)

**Response:** We have revised the sentence.

**C 6**. Intro- the link between DM-Smoking-HS remains elusive and why this is the focus of the study is still unclear from the literature reviews done.

**Response:** Thank you for your valuable comment. Both DM and smoking are recognized risk factors for HS, but their combined influence on HS development remains insufficiently studied. Smoking, as a modifiable behavior, may exacerbate the systemic inflammation and metabolic dysregulation associated with DM, potentially increasing the risk of HS. Despite this plausible mechanism, studies explicitly examining this triad are scarce. Therefore, we aimed to investigate how changes in smoking behavior affect the risk of HS in patients with DM, using a large-scale, nationally representative claims database in Korea. By addressing this gap, our study seeks to clarify the role of smoking intensity in HS development and provide insights into modifiable risk factors, particularly in patients with comorbid DM. Our goal is to provide insights into actionable strategies to mitigate HS risk in this high-risk population. We have supplemented the contents in **P5 L79-82.**

**C 7**. Materials & methods:
- need proofreading by a native speaker who are familiar with health related research manuscript.

**Response:** As you recommended, proofreading had been done.

**C 8**. Materials & methods:

- some confounding factors not explored: anti-inflammatory medication, surgical procedures, other medications/ treatment for HS, and diabetic control (HBA1c levels) need to be mentioned.

**Response:** Thank you for your insightful comments. However, what we aimed to investigate was the risk of HS development among groups classified by smoking behavior in patients with DM, not the outcomes after HS has already occurred. (**P5 L97-98**; We excluded patients who had already been diagnosed with HS.) Therefore, HS treatment or surgical procedures may be of limited relevance to the primary focus of this study.

Although HbA1c was not measured and therefore not observed, we adjusted for indirect indicators such as fasting glucose, DM duration, OHA use, and insulin use.

The use of anti-inflammatory medication (for medical reasons other than HS) was considered unlikely to be a significant factor affecting the development of HS itself, as these medications are primarily prescribed for pain management after HS onset.

However, we did acknowledge the potential limitations of this observational study in adjusting for confounding factors in the limitations section **P12 L266**.

**C 9**. Materials & methods:

- Definition of smoking intensity change: suggest to change to definition of a change in smoking intensity

**Response:** We have changed it. Thank you for your comment.

**C 10.** Materials & methods:

- Definition of never, former and current smokers were unclear, reducers, sustainers, increasers and starters? These definition may be better presented in a table. Would be good to get this presented as part of an experimental design flowchart (as a figure)

**Response:** Thank you for your valuable comment. The terms 'never,' 'former,' and 'current' smokers mentioned in **P6 L120-121** and in **Table 2** refer to classifications based on each status at the initial and follow-up time points. In contrast, the seven categories (e.g. quitters, 50% reducers, etc.) described at **P6 L123-133** and in **Tables 1, 3, and 4** represent groups classified based on **changes** observed **between** the initial and follow-up time points. These seven categories are summarized and presented in **Figure 2**.

**C 11**. Materials & methods:

-line 126: All blood samples were taken during fasting

**Response:** The content has been revised. (**P7 L138**)

**C 12**. Results
- line: 145 The cohort comprised of 1,705,427 participants (581,618 males [34.1%], 1,123,809 females [65.9%]; mean [SD] age, 59.03 [11.76] years). (added of)

**Response:** Thank you for your comment. The content has been revised. (**P8 L164**)

**C 13**. Results
- line 165: Kaplan-Meier estimates showed that the incidence of HS was higher in increasers than in quitters. (Figure) (which figure? Each figure should have a number assigned)

**Response:** Since new figures have been added, the previous “Figure” has been renamed as “**Figure 3**”.

**C 14**. Discussion
- Minor proofreading help with grammar such as putting connectors where appropriate

**Response:** Thank you for your comment. We have carefully revised the manuscript to address minor grammatical issues and added appropriate connectors to improve the flow and coherence of the text.

**C 15**. Discussion
- Would be good to discuss the effects of polyaromatic carbohydrates and nicotine in HS development to better link tobacco smoking and HS

**Response:** Thank you for your valuable comment. The effects of polycyclic aromatic hydrocarbons (PAHs) and nicotine on the development of HS, and their role in linking tobacco smoking to HS have been summarized in the discussion section. Key mechanisms include the activation of the aryl hydrocarbon receptor (AhR) by PAHs, promoting chronic inflammation and keratinocyte dysfunction, and the contribution of nicotine to follicular plugging, immune suppression, and increased susceptibility to bacterial invasion.

**C 16**. Discussion
- Would like to suggest the authors to refrain from using first-person language in the discussion to keep the discussion more impersonal.

**Response:** Thank you for your suggestion. We have revised the discussion section to avoid the use of first-person language in order to maintain an impersonal and objective tone.

**C 17**. - Limitation: HS treatment can be many, and over the course of 2 years, participants may have taken multiple medications to prevent or treat the disease which may lead to bias in the findings. Medication data was not available and should be mentioned in the limitation.

**Response:** Thank you for your valuable comment. The response to this comment can be framed similarly to **C8**. The use of treatment medications for HS is not directly related to the focus of our study. Furthermore, no medications have been definitively proven to prevent HS to date, which limits the inclusion of such information in this paper. However, we acknowledge that there may be confounding factors not considered in our analysis, and we have added this as a limitation in the revised manuscript (**P12 L266)**.

**C 18**. Tables and figures:
- suggest to refer to "figure" as "figure 1"

**Response:** Since new figures have been added, the previous “Figure” has been renamed as “**Figure 3**”.

**C 19**. Tables and figures:
- suggest to increase figure 1 image quality to 300 dpi

**Response:** Thank you for your advice. The image quality of **Figure 3** (renamed from ‘figure 1’) is 330 dpi.

**C 20**. Tables and figures:
- suggest to include experimental design flowchart in the form of a figure

**Response:** Thank you for your suggestion. We have created a flowchart and included it as **Figure 1**, detailing the number of individuals at each stage of the study, including those potentially eligible and the reasons for non-participation at each stage. The seven categories (classified based on **changes** observed **between** the initial and follow-up time points) are summarized and presented in **Figure 2**.
