# Supplementary material for: A link between smoking behaviors and the risk of hidradenitis suppurativa in diabetic patients
Source: PLoS One. 2025 Jun 5;20(6):e0325357. doi: 10.1371/journal.pone.0325357 (PMC12140424; doi:10.1371/journal.pone.0325357)
Supplement: Attachment — Submitted filename: Response to Reviewers_2nd revision.docx [file pone.0325357.s003.docx]

To

Editor of PLOS ONE

Submission ID d914ed04d190a2b4

"Hidradenitis suppurativa (HS) in diabetes mellitus patients: A link between alterations in smoking intensity and the risk of developing HS"

Thank you very much for your kind editorial letter.

We have attempted to carefully and thoroughly address all concerns raised by the editors and reviewers. With the help of your suggestions, we believe our manuscript has significantly improved.

We trust that we therewith have fulfilled all the Editor’s and Reviewer’s requests.

Thank you very much for your consideration.

Sincerely,

Ji Hyun Lee, MD. PhD

Department of Dermatology, Seoul St. Mary`s Hospital, College of Medicine, The Catholic University of Korea, Seoul, 06591, Korea.

Tel: +82-2-2258-1398

Fax: +82-70-7382-1380

E-mail: [yiji1@hanmail.net](mailto:yiji1@hanmail.net)

**Responses to Reviewer**

Reviewer(s)' Comments**(C)** to the Author:
**Reviewer: 1**

Comments to the Author

**C 1.** -Add reference for the Korean Association of Laboratory Quality Control guidelines.

**Response:** Thank you for your suggestion. We have added an appropriate reference to support the guidelines provided by the Korean Association of External Quality Assessment Service (KEQAS), which is the official and accredited national body responsible for external quality assessment in Korea. The reference has been cited in the revised manuscript as follows:

Kim S, Lee K, Park HD, et al. *Schemes and Performance Evaluation Criteria of Korean Association of External Quality Assessment (KEQAS) for Improving Laboratory Testing.* Ann Lab Med. 2021;41(2):230–239. <https://doi.org/10.3343/alm.2021.41.2.230>

**C 2.** -Please add the possible explanation for finding no association after BMI stratification to the discussion section as well.

**Response:** The lack of association between changes in smoking intensity and the risk of HS after stratifying by BMI could be attributed to several factors. The limited number of HS events in the study may have reduced the statistical power to detect differences across BMI categories, increasing the likelihood of a Type II error.

Also, if the BMI distribution in the study was relatively homogenous or skewed toward specific categories, it might have limited the ability to observe differences between groups. And the interplay between smoking, BMI, and HS risk is complex and likely involves biological pathways that are not fully elucidated. Factors such as inflammation, hormonal changes, or metabolic syndromes linked to both smoking and BMI might obscure direct associations. Further research with larger and more diverse cohorts is necessary to clarify these findings and explore the interaction between smoking, BMI, and HS risk. We added it in **P 12 L 254-265.**
